# Draft Genome Sequence of Endophytic Bacterium *Enterobacter asburiae* PDA134, Isolated from Date Palm (*Phoenix dactylifera* L.) Roots

**DOI:** 10.1128/genomeA.00848-16

**Published:** 2016-08-18

**Authors:** Mahmoud W. Yaish

**Affiliations:** Department of Biology, College of Science, Sultan Qaboos University, Muscat, Oman

## Abstract

In this report, a draft of the *Enterobacter asburiae* strain PDA134 genome was sequenced. This bacterial strain was isolated from the root tissue of a date palm, where it has the ability to produce 1-aminocyclopropane-1-carboxylic acid (ACC) deaminase and indole-3-acetic acid (IAA) under salinity stress.

## GENOME ANNOUNCEMENT

Endophytic bacteria have the ability to provide host plants with their required nutrition and phytohormones (1). These bacteria can enhance the growth of the plant through the synthesis of different growth-promoting substances, such as the indole-3-acetic acid (IAA) phytohormone and the 1-aminocyclopropane-1-carboxylic acid (ACC) deaminase, ethylene’s direct precursor, which releases a gaseous stress hormone involved in plant senescence and programmed cell death under stress (2, 3).

In this work, a draft genome sequence of *Enterobacter asburiae* strain designated PDA134 was reported. This strain was previously described based on functional characterization and the biochemical products with relation to plant growth promotion (4). Initially, this strain was identified as *Klebsiella oxytoca* based on partial 16S rRNA gene sequencing; however, the near-complete genome sequence showed that the greatest hit length was similar to that of the *Enterobacter asburiae* and *Enterobacter cloacae* species. In this report, the *Enterobacter asburiae* PDA134 was identified based on 16S rRNA, RNA polymerase β subunit (*rpoB*), DNA gyrase (*gyrB*), initiation translation factor 2 (*infB*), and ATP synthase β subunit (*atpD*) gene sequence analyses, as previously described (5).

The genome was sequenced using the DNA paired-end library method carried out at the DNA sequencing facilities at the BaseClear Company, The Netherlands, as a service provider. Briefly, the genomic DNA (gDNA) was fragmented, and DNA adapters were ligated to both ends of the DNA fragments. The library was then sequenced on the Illumina HiSeq 2500 sequencer. The sequence reads were filtered and trimmed based on Phred quality scores. The analysis was carried out using the *de novo* assembly option of the CLC Genomics Workbench, version 7.0.3. The optimal k-mer size was automatically determined using KmerGenie (6). The contigs were linked and placed into scaffolds or supercontigs. The analysis was carried out using the SSPACE Premium scaffolder, version 2.3 (7). The gapped regions within the scaffolds were (partially) closed in an automated manner using GapFiller, version 1.10 (8). DNA sequences were annotated using the Prokka Prokaryotic Genome Annotation System, version 1.6 (Victorian Bioinformatics Consortium).

The results showed that the *Enterobacter asburiae* PDA134 genome consisted of 4,699,624 bp, assembled into 26 scaffolds ranging in length from 900,290 to 328 bp, with a G+C content of 56.15%. There were 4,352 putative coding regions, including 3,310 genes with a known function representing 1,549 enzymes mapped on 2,987 Kyoto Encyclopedia of Genes and Genomes (KEGG) pathways. The gene list also contained 69 tRNA genes and two rRNA clusters.

The announced endophytic bacterial genome in this project encoded a wide-host-range VirA protein (9), a nitrogen fixation (VnfA) protein (10), tryptophan synthase alpha and beta chains, which may involve IAA synthesis in bacteria (11), an ACC deaminase/d-cysteine desulfhydrase (12), several putative siderophore biosynthesis, binding, and transport proteins (13), multiple antibiotic resistance proteins (MarR) (14), ampicillin-resistant β-lactamase proteins (15), and a streptomycin biosynthesis protein (StrI) (16). The presence of these genes might promote plant-microbe communication and symbiosis.

### Accession number(s).

This whole-genome shotgun project has been deposited at DDBJ/ENA/GenBank under the accession no. LSQV00000000. The version described in this paper is version LSQV01000000.
